# Selected Principles of Pankseppian Affective Neuroscience

**DOI:** 10.3389/fnins.2018.01025

**Published:** 2019-01-17

**Authors:** Kenneth L. Davis, Christian Montag

**Affiliations:** ^1^Pegasus International, Inc., Greensboro, NC, United States; ^2^Department of Molecular Psychology, Institute of Psychology and Education, Ulm University, Ulm, Germany; ^3^MOE Key Lab for Neuroinformation, The Clinical Hospital of Chengdu Brain Science Institute, University of Electronic Science and Technology of China, Chengdu, China

**Keywords:** Jaak Panksepp, affective neuroscience, affective neuroscience personality scales, subcortical, cross-species, affects, primary-process emotions, emotion

## Abstract

In the early nineties of the twentieth century Jaak Panksepp coined the term “Affective Neuroscience” (AN) today being accepted as a unique research area in cross-species brain science. By means of (i) electrical stimulation, (ii) pharmacological challenges, and (iii) brain lesions of vertebrate brains (mostly mammalian), Panksepp carved out seven primary emotional systems called SEEKING, CARE, PLAY, and LUST on the positive side, whereas FEAR, SADNESS, and ANGER belong to the negative affects. Abundant research into human clinical applications has supported the hypothesis that imbalances in these ancient primary emotional systems are strongly linked to psychiatric disorders such as depression. The present paper gives a concise overview of Panksepp's main ideas. It gives an historical overview of the development of Panksepp's AN thinking. It touches not only areas of neuroscience, but also shows how AN has been applied to other research fields such as personality psychology. Finally, the present work gives a brief overview of the main ideas of AN.

## A Brief Introduction To Jaak Panksepp's Scientific Career

The scientist who coined the term *affective neuroscience*, Panksepp (1991, 1992), had the insight as a young clinical psychology student working in a mental hospital that understanding emotions was the key to developing more effective treatments for psychiatric hospital patients and all those suffering with psychopathology. This insight led to his graduate school career change from clinical psychology into what we now call neuroscience. He had also realized that the level of understanding that was needed would require brain research that could not be conducted on human beings. Hence, he began probing the neural constitution of emotions in the deep foundations of the mammalian brain. In his dissertation he was able to use electrical stimulation of the brain (ESB) to elicit two distinct emotional attack behaviors in rats: “affective attack” toward another rat and a predatory “quiet bite” attacking a mouse. He was further able to show that these rats subjectively experienced these two contrasting emotions, meaning they would work to turn off the stimulus eliciting the affective RAGE attack but would work to turn on and receive more of the stimulus eliciting the “quiet bite” attack, which was later shown to be activating the SEEKING system.

In the wake of the discovery in the early 1970s of endogenous opioids in the mouse brain, Panksepp began working on another potential emotional behavior system in the brain. J. P. Scott, a senior colleague at Bowling Green State University (BGSU), had studied the social behavior of dogs for many years (Scott and Fuller, 1965) and was currently exploring separation distress vocalizations in puppies. Meanwhile, Jaak had recognized similarities between opioid withdrawal in drug addicts and the social distress caused by broken relationships and had also noticed that opioid addicts frequently came from marginal family social backgrounds. Panksepp hypothesized that opioids might be related to mammalian separation distress calls, and a BGSU research group soon demonstrated that low doses of morphine would soothe the separation distress vocalizations in canine puppies (Panksepp et al., 1978). In 1980, after his lab had successfully mapped distress vocalization sites in the guinea pig brain, Panksepp went on to publish his “opioid hypothesis” (Panksepp et al., 1980), namely, that brain opioids likely underlie the formation of social attachments and modulate social emotions and behaviors.

By 1982, Panksepp was convinced that there were at least four biological brain-based emotional action systems (Panksepp, 1982), which at that time he labeled Expectancy, Rage, Fear, and Panic. In a *Psychological Review* paper (Panksepp, 1992) and especially with the publication of *Affective Neuroscience* (Panksepp, 1998), Panksepp had expanded his list of primary emotions to seven well-documented primary-process emotional command systems (SEEKING/Expectancy, RAGE/Anger, FEAR/Anxiety, LUST, CARE/Nurturing, PANIC/Sadness, and PLAY/Social Joy) and introduced the use of capitalization to distinguish these primary emotional brain systems from the use of his chosen emotion labels in common language. The mapping of the seven primary emotional systems by means of electrical stimulation of the mammalian brain including pharmacological challenges and brain lesions represents the heart of what Panksepp named affective neuroscience. Of note, Panksepp concluded that there was insufficient evidence to include Social Dominance as a primary emotion, and he considered it an acquired behavior [see further thoughts on this in van der Westhuizen and Solms (2015)]. Panksepp did include a detailed treatment of the homeostatic affect HUNGER in *Affective Neuroscience* but did not consider it in the same category as emotional affects, which were more directly relevant to mammalian psychopathology and personality.

The remainder of this review essay will outline key themes of affective neuroscience as developed in the research and writings of Jaak Panksepp. Rather than continuing to review the development of his thinking throughout his scientific career, the focus here will be on selected affective neuroscience principles first featured in *Affective Neuroscience* but also elaborated in *The Archaeology of Mind* (Panksepp and Biven, 2012), *The Emotional Foundations of Personality* (Davis and Panksepp, 2018), as well as numerous theoretical review papers.

## Mammals Are Deeply Affective

All mammals are sentient beings meaning that it feels like something to be alive and dealing with the challenges in their worlds. The philosopher, Langer, in her book, *Mind: An Essay on Human Feeling*, writes “*To feel is to do something*” (Langer, 1988, p.7). The word “emotion” is derived from the Latin verb “emovere” meaning “to move out,” and that seems to be what we observe already in the evolution of early life. Primal emotions and their accompanying affects appear to have acquired the capacity to move animals to action in ways that promoted their survival. Emotions prodded animals to explore for resources (SEEKING), compete for and defend those resources (RAGE/Anger), escape from and avoid bodily danger (FEAR), and identify potential mates and reproduce (LUST). Then, mammals with their more social orientation acquired the motivational system for nurturing their offspring (CARE); the powerful separation distress system for maintaining social contact and social bonding (PANIC/Sadness); and the complex system stimulating especially young animals to regularly engage in physical activities like wrestling, running, and chasing each other (PLAY/Social Joy), which helps them bond socially and learn social limits and which seems to carry over into the “ribbing” and joking that continues to add fun in adulthood. Evolution has endowed mammalian brains with at least these seven primary-process emotional action systems, which serve as survival guides. These primary emotions arise from subcortical brain regions that are largely homologous, especially across mammals, with each emotion having a distinct brain anatomy, neuropharmacology, and physiology (for details beyond the scope of this paper, see Panksepp, 1998; Panksepp and Biven, 2012). Jaak Panksepp felt that the key affective neuroscience question was the neural constitution of raw affects (Panksepp et al., 2017, p.206), which was essential for understanding our own affects and for developing better psychiatric treatments for emotional imbalances but which would require further causal preclinical research into our ancestral subcortical primary-process emotional brain systems. For a recent published obituary on Jaak Panksepp's life please see Davis and Montag (2018).

## Ancestral Voices

Mother Nature (aka evolution) speaks to all mammals in the oldest language, the language of emotional affects. The ancestral voices (to use Ross Buck's phrase) guide their choices as they navigate life. Each of the primal emotional affects is evaluative, that is, has a valence that is either pleasant or aversive and signals objects or situations to approach in the case of the pleasant ones (SEEKING, LUST, CARE, and PLAY) or to avoid in the case of the aversive ones (RAGE, FEAR, and PANIC).

Yet, experiencing a primary affect does not necessarily mean all mammals can self-reflect on their emotional experiences. That capacity may be reserved for the more cortically endowed mammals. However, “raw” primary affects are experienced as pleasant or aversive qualia, which alter behavior and provide for secondary-level learning (conditioning principles). The emotional minds of most mammals may be limited to displaying “intentions-in-action” rather than a more reflective “intentions to act.” In Endel Tulving's terminology, the capacities of most mammals likely combine anoetic (without knowing) and noetic (knowing) consciousness without necessarily attaining autonoetic consciousness—being able to sufficiently hold experiences in memory to review the past and anticipate the future (Tulving, 1985). We know that animals experience primary affects because of empirical measures: They will work vigorously to sustain affective states by learning to turn on ESB evoking positively valenced emotions and correspondingly escape or avoid the negatively valenced emotions. They will also demonstrate conditioned place preferences or aversions for situations where they have experienced such stimulation in the past (Panksepp, 2011). Further, animals will emit conditioned positive and negative vocalizations in places where they have experienced positively or negatively valenced ESB (Knutson et al., 2002). However, we are unable to measure feelings (affective qualia) directly, not even in humans.

Apart from experiencing primary-process emotional affects, what is much more difficult to study in the non-human mammalian world is Tulving's autonoetic consciousness characterized by the capacity of humans to experience affective nuances often reflecting human higher-order cognitions and language including having thoughts about thoughts. This represents a limitation of cross-species affective neuroscience. However, subtler models of affective concepts such as pessimism in dogs (Mendl et al., 2010), optimism in rats (Rygula et al., 2012, 2015), and regret in rats (Steiner and Redish, 2014) are appearing in cross-species studies.

## Primordial Emotions Are Innate But Are Also Adaptive Learning and Memory Systems

The primary-process emotions require no learning. It is not necessary to teach a child to become angry, fearful, or to panic after having lost sight of parents in a crowd. Nor do we need to teach children how to play. These evolved foundational tools for living are somehow automatically built into our heritage. However, these evolutionarily/genetically endowed primary-process emotional brain systems are not fixed functions but are able to learn and adapt to novel environmental experiences throughout the life of an individual. Indeed, as introduced above, the valenced affects associated with each of the primary emotions serve as endogenous rewards and punishments for behaviors that activate emotions. For example, receiving painful stings from hornets flying out of the nest you accidently disturbed fills you with fear, and without thinking about it you immediately react by running away from the menace. Having reached a safe distance, you feel relief that you seem to be out of danger, and likely begin examining the tiny wounds, which are beginning to swell slightly as the pain intensifies, and you may begin to clarify (at a safe distance) the details of the hornet nest's appearance and location. This event will be forever embedded in your memory, and you will have learned to avoid repeating this experience by remaining more vigilant when outdoors walking through unfamiliar terrain.

Each primary-process emotional command system likely encompasses a separate reward or punishment system. These learning systems can be thought of as secondary-processes integrating new experiences into the primary framework allowing for previously neutral environmental stimuli to elicit the emotion and for novel reactions to become associated with such stimuli. For an example of novel reactions to an emotional arousal, over the ages, humans when threatened have learned to reach for their swords, and more recently their pistols instead of clenching their fists. However, more evidence is needed regarding the extent to which these different emotional systems encompass different learning and memory parameters.

While deep brain stimulation (DBS) allows researchers to demonstrate that brain stimulation at specific sites evokes distinct emotional behaviors that are accompanied by corresponding affects, there remains the question of whether the emotional affects elicited at these sites are similarly distinct. We do know that rats can learn to discriminate between DBS in the hypothalamus and septal regions of the brain (Stutz et al., 1974). We also know that animals can distinguish between the emotional states induced by the addictive drugs morphine and cocaine (Overton, 1991). We also know that DBS in humans at homologous brain sites seems to evoke homologous affects (see Panksepp, 1985 for a review). However, much more research needs to be done before there is any clear assurance regarding the number of distinct primary affects or the role of electrical or pharmacological stimulants in generating those affects.

## Primary-Process Emotions Survive Decortication

Primary affects are constituted at the subcortical level. That is true for primary emotional affects such as ANGER and FEAR, or homeostatic affects such as HUNGER and THIRST. There is ample evidence that primary-process emotional brain systems do not require the neocortex. In rats, decortication does not block the rewarding effects of subcortical ESB (Huston and Borbely, 1973, 1974). In humans, strong emotions decrease cortical activation (Damasio et al., 2000) and cortical damage often leads to increased emotionality, which is consistent with the cortex generally providing the inhibition or regulation of emotions rather than activation (Liotti and Panksepp, 2004).

Further support in humans for subcortical emotions without cortex is offered by Merker (2007) who has reviewed the case of hydranencephalic children who are born without a cerebral cortex. He writes that even without a cerebral cortex, these children “express pleasure by smiling and laughter, and aversion by ‘fussing,' arching of the back and crying (in many gradations), their faces being animated by these emotional states” (Merker, 2007, p.79). He further comments that their “[emotional] behaviors are accompanied by situationally appropriate signs of pleasure or excitement on the part of the child” (Merker, 2007, p.79). These children clearly show that appropriate emotional responses even in humans do not require the participation of the neocortex. As further evidence of an independent subcortical brain that can function without a neocortex, Merker also reviewed Penfield and Jasper (1954) in which brain surgery under local anesthesia was performed on conscious patients with a history of severe epileptic seizures. These surgeons removed sizeable sectors of cortical tissue while communicating with the patient, which “never interrupted the patient's continuity of consciousness even while the tissue was being removed” (Merker, 2007, p.65).

Along these lines, Damasio et al. (2013) reported the case of Patient B. who had contracted Herpes Simplex Type I encephalitis (HSE), which had destroyed much of his cortical brain tissue with extensive bilateral cortical damage including temporal lobe and temporal pole cortices, posterior orbitofrontal cortices, and the anterior cingulate cortices. Plus the left and right insular cortices were entirely destroyed (including the insula itself) as well as the bilateral entorhinal cortex, hippocampus proper, and amygdala.

In short, Patient B. had lost structures thought by many to be integral to emotional experience: the insula (Craig, 2011), the amygdala (LeDoux, 2012), and the anterior cingulate cortex (Bijanki et al., 2015). Yet, all indications (including the observations of strangers, the observations of the research team, psychological evaluations, and a structured questionnaire completed by his spouse comparing his emotional behavior before and after his disease) were that Patient B. retained a full range of appropriate emotions after his brain disease.

The lack of importance of the cortex for emotional behavior and displays was supported by Whishaw's (1990) review of the experimental rat decortication literature. While there were differences between whether the surgery took place neonatally or closer to maturity, the neonatal group showed few deficits. With neonatally decorticated rats, loss of cortex essentially did not disrupt survival/emotional behaviors and displays. These subjects exhibited no interruption in post-surgery sucking and grew to maturity with near normal weights. They exhibited normal posture during face washing with no deficits in grooming. They were able to reproduce with six out of eight females being able to successfully raise their litters with normal cleaning, suckling, and caring for their pups. As juveniles, they played as much as controls, and most components of aggressive and defensive behavior were present including lateral displays in response to an intruder and conditioned “freezing” after having been attacked as an intruder and later being reintroduced as the intruder to the same cage with the same resident.

Panksepp's group replicated the effect of neonatal decortication on rat juvenile play (Panksepp et al., 1994). Measures of play vigor and tests of play solicitation behaviors did not detect differences, which suggested that play motivation was intact in the decorticate rats. They did observe a decrease in frequency of pinning and shorter pin duration. However, additional control studies suggested that these changes were likely due to motor changes and reduced somatosensory sensitivity. In short, they found that the play of decorticate rats appeared normal.

Indeed, 16 graduate students were asked to observe a pair of juvenile rats for 30 min, one of which had its cortex surgically removed neonatally and the other, a control subject, that only had received sham surgery, and to decide which one had been decorticated. Most chose the control rat pup with an intact cortex (Panksepp, 2015). Overall, Panksepp concluded, like Whishaw, that “the results generally indicate little participation of the neocortex in the instigation of rough-and-tumble play” (Panksepp et al., 1994, p. 429).

By contrast this group reported that much smaller thalamic lesions had greater influence on play in rats. Lesions of the parafascicular region of the thalamus reduced pinning by 73% but also reduced play solicitation behavior, likely indicating decreased play motivation compared with controls showing the lesion effects were specific to play (Siviy and Panksepp, 1985).

The theme of small subcortical lesions having dramatic influences on emotional behavior such as losing virtually all spontaneous activity after ablating the periaqueductal gray (Bailey and Davis, 1942) was convincingly addressed by Fernandez de Molina and Hunsperger (1962). They showed that the rage responses of cats could be evoked by ESB along the basic subcortical mammalian RAGE system running from the periaqueductal gray (PAG), at the lowest level up to the medial hypothalamus and on up to the medial amygdala at the highest level in decreasing levels of importance. As such, they found that aggressive responses evoked by ESB of the amygdala were abolished by lesions at the level of the hypothalamus or PAG. Aggressive responses from the hypothalamus were dependent on the PAG but not on the amygdala. And, at the lowest level, aggressive responses evoked at the PAG level were not dependent on either of the higher two levels. Further, field studies with cats receiving small lesions to what they called the “hissing zone” of the PAG, “when confronted with a dog, no longer hissed or attacked” (Fernandez de Molina and Hunsperger, 1962, p. 201).

Clearly, our primary-process emotions and their powerful affective messages are deeply embedded in our mammalian brains. Humans and other mammals still experience these emotions without a neocortex, and the subcortical regions are organized in an evolutionary hierarchy of importance. Understanding these cross-species emotional systems may represent the greatest challenge to neuroscience. Again, in the words of Jaak Panksepp, “For A[ffective] N[euroscience] the key question is the neural constitution of raw emotional, homeostatic and sensory affects” (Panksepp et al., 2017, p. 206).

## The Neocortex Is Essentially a Blank Slate at Birth

The Pankseppian affective neuroscience view is that “the neocortex is fundamentally tabula rasa at birth,” Latin for “blank slate” (Panksepp and Biven, 2012, p.427), and it is through experience that the neocortex is “programmed” (likely through interactions with subcortical regions) to acquire its capacities that as we reach maturity come to seem like “hard-wired” brain functions. But, is any function in neocortex genetically determined? It might seem to many that the visual cortex is a possible candidate. Yet, Sadato et al. found otherwise (Sadato et al., 1998). They found that people naturally blind from an early age provided an example of occipital cortical regions that had been “programmed” to perform a non-visual function and supplanting the somatosensory area. Specifically, positron emission topography (PET) showed that when individuals who were blind from an early age were performing a braille task, the tactile processing that would normally occur in conventional somatosensory cortex had been shifted to areas in the occipital cortex that are normally assumed to process visual stimuli.

Another PET study provided evidence from blind subjects for the use of visual cortex for auditory processing. Weeks et al. (2000) used PET to determine which cortical areas were being activated during an auditory localization task. While sighted and blind subjects both showed activity in the posterior parietal cortex, only blind subjects also showed activity in the occipital cortex, a cortical area that is normally associated with visual processing. Thus, cortical plasticity seems to allow blind individuals to develop enhanced tactile and auditory capabilities by redirecting neocortical regions typically thought of as visual processing regions to enhance other sensory functions.

Experimental research from Mriganka Sur's group at MIT (von Melchner et al., 2000), supported the “reprogramming” findings documented in blind subjects. Using ferrets (a species born in an exceptionally immature stage), visual input was surgically redirected to auditory cortex shortly after being born, and as predicted the auditory cortex was developmentally programmed to process vision: A follow-up study using similar procedures with mice showed that rewired mice could learn visually-cued conditioned fear (Newton et al., 2004). Both sets of animals with visual input redirected to auditory cortex developed fine cortical visual abilities even though the neocortical processing was developmentally constructed rather than genetically dictated.

That we should not expect to find evolved specializations in the neocortex was further supported by the report in *Science* that a single gene, called *ARHGAP11B*, was responsible for much of the massive expansion of the human neocortex. The researchers further determined that this newly identified gene was found only in humans, Neanderthals, and Denisovans—another extinct hominid line in southern Siberia. The gene was not found in our closest living evolutionary relative, chimpanzees (Florio et al., 2015). This finding makes it increasingly unlikely that any of our neocortical higher mental abilities represent evolutionary genetically-determined specializations like are found in subcortical brain regions.

## The Neocortex Is Likely Organized by the Subcortical Functions of the Brain

The idea, that subcortical systems guide the development of cortical specializations gained support from Panksepp's group investigating the possible role of play in the development of the frontal cortex. Working on evidence that right hemisphere frontal lobe deficits were associated with Attention Deficit/Hyperactivity Disorder (ADHD) and the knowledge that the symptoms of frontal lobe damage generally resemble ADHD, it was hypothesized that right frontal lobe damage might be a useful rodent model of ADHD. They found that rat right frontal lobe lesions (performed at three or four days of age) significantly increased playfulness (as measured by pins and dorsal contacts) as well as increasing activity levels confirming the hypothesis (Panksepp et al., 2003) [Note: A replication obtained the same results from lesions to the left or right frontal lobes; please see also a new work on individual differences in tendencies toward ADHD and primary emotional systems as assessed with the Affective Neuroscience Personality Scales by Wernicke et al. (2018)].

Activity levels of both sham and lesioned animals decreased with age. However, in the rat pups with frontal lesions, 1 h per day of “play therapy” for seven consecutive days (tested at 30 days of age) significantly reduced the number of pins and overall activity, “ameliorating” the elevated play urges and activity levels. Indeed, the play therapy even significantly reduced overall activity in the sham lesion control subjects.

Additional tests in new subjects that had no lesions indicated that play experiences also increased behavioral inhibition not specific to increased fear. Combining both experiments, the authors suggested “the possibility that one of the long-term functions of social play is to promote maturation of various higher brain areas, including frontal cortical ones” (Panksepp et al., 2003, p.103–104). By some little understood means, an activated subcortical PLAY system seems to be facilitating the development of the frontal lobe behavioral inhibition function including a more mature regulation of excessive play urges perhaps also reflected in undesirable impulsivity.

Another line of research proposing that innate subcortical systems guide the development of refined neocortical capacities comes from the work of Mark Johnson's group linking infant facial preferences to adult face recognition and related capacities. Johnson's work also started with animal research: the laboratory imprinting of newly hatched domestic chicks (Horn, 1985; Horn and Johnson, 1989). Johnson and Horn (1988) reported that newly hatched chicks were *predisposed* to follow and attend to the *face* of an imprinting object with the correct arrangement of facial features including the heads of models and other bird species. They determined that the subcortical optic tectum of the chick brain, which is homologous to superior colliculus of the mammalian brain, was likely the source of this neonatal bias. However, regardless of the quality of the imprinting object, chicks were capable of developing strong preferences for the imprinting objects over novel stimuli, a learning process that was dependent on chick forebrain (homologous to mammalian cortex). The initial chick predisposition and the acquired preference were hypothesized to represent independent brain systems. That is, selective cortical lesions impaired the acquired preferences but not the predisposition (Johnson and Horn, 1986). It was further hypothesized that the subcortical predisposition system guided or “tutored” the cortical system in acquiring information about the mother hen or surrogate perhaps by a process as simple as a predisposing bias that oriented the chick toward the hen or appropriate substitute (Johnson et al., 2015, p.170–171).

This animal model was extended to primate and human research confirming a superior colliculus/pulvinar/amygdala system as the likely source of neonatal face processing predispositions with the superior colliculus and pulvinar receiving direct retinal input allowing for rapid orienting to faces and direct eye contact, and the detection and processing of facial threat expressions. They proposed that this subcortical “fast-track” pathway facilitates detecting eye contact and the orientation toward and processing of faces from infancy through adulthood with the prefrontal cortex providing top-down modulation but also involving additional key structures such as the fusiform gyrus, superior temporal sulcus, and orbitofrontal cortex in a two-way model that added developmental complexity to the acquired coordination of social facial processing (Senju and Johnson, 2009).

The previous two research lines illustrate that basic subcortical processes likely constitute primary-process experiences that become developmentally elaborated in the neocortex (Panksepp and Biven, 2012). With maturation, these physically as well as evolutionarily separate brain regions develop a reciprocal seesaw-like relationship to weigh whether a life event should trigger or inhibit the expression of a primary emotion with imbalances in either direction potentially becoming dysfunctional (Liotti and Panksepp, 2004). It has also been reported that more ancient cortical midline structures such as the ventral medial prefrontal cortex may participate in such reciprocal relationships with more cognitive brain regions such as the lateral/dorsal prefrontal cortex (Goel and Dolan, 2003; Northoff et al., 2004). However, despite increasing cortical influence with maturation, it remains clear that subcortical emotional systems retain the capacity to shut down cortical activity during strong emotional experiences (Damasio et al., 2000).

Along these lines, there is evidence that all long-term memory has an emotional component, and with stronger emotions, the resulting memory becomes correspondingly stronger with the exception that traumatic emotional experiences can reduce memory retrieval and even cause amnesia (Alberini, 2010). Typically, stronger emotional activation is associated with stronger memory retrieval with strong positive emotional experiences such as a wedding and strong negative emotional experiences such as the funeral of a loved one both enhancing memory strength. Moreover, one of the basic functions of the primary-process emotional memory systems may be arousal and the associated drawing attention to specific events that can facilitate the formation of memories for important life events that in turn can subsequently inform how we respond to future life events.

With the ongoing guided acquisition of experience, the neocortex surely provides many refinements and expansions of our basic subcortical capabilities with the caveat that an ever vigilant subcortical brain can assert its predominance in response to genetically-linked wisdom embodied in, e.g., pent up urges to play or fear engendered by the menacing face of a predator. Hence, if an evolutionary significant cue resonates in the primary emotional systems, the subcortical energy can override our activity in the cortical thinking cap. This developed two-way (bottom-up and top-down) relationship requires the ongoing involvement of our affective states influencing our perceptions, thoughts, and urges as well as the acquired cortical capacity to modulate our emotional evolutionary foundations in order to achieve an appropriate seesaw positioning for ongoing life events. Yet, much research remains to be done to illuminate the mechanisms involved in these evolutionarily integrated processes.

## The Route to Discovering Primary-Process Emotions

At least since the ancient Greeks first speculated about the four humors and Ptolemy proposed the Sun, Moon, and planets set our behaviors in motion, humans have speculated about the basis of personality and psychopathology. More modern theories have ranged from Freud's theory of sex and aggression on personality development (Freud, 1920/1990) to the thesis that the differences in our personalities are mostly learned (Miller and Dollard, 1941).

Cattell pioneered using the lexical hypothesis—the assumption that any important human characteristic was embedded in language—and factor analysis and first reported four personality factors (Cattell, 1933), but as increasing computing power allowed for working with larger data sets, he eventually proposed as many as 19 distinct factors with 16 being used in his most widely used personality assessment, the 16PF (Cattell et al., 1970). However, others using factor analysis reported as few as three (Eysenck, 1967) and as many as 20 (Jackson, 1974) personality dimensions. Many others took up Cattell's approach considering factor analysis as a more theory-free and objective statistical approach to parsing personality into its components, an approach that may have peaked in accepting five personality dimensions (Tupes and Christal, 1992 – originally reported in 1961). The “Big Five“ were massively shored up by Lewis Goldberg's seemingly exhaustive factor analytic studies (Goldberg, 1990, 1992) as well as a report confirming these five personality dimensions plus Dominance in chimpanzees (King and Figueredo, 1997). The Big Five scales are typically labeled Extraversion, Agreeableness, Conscientiousness, Emotional Stability, and Openness to Experience. Even the psychiatric world got on the Big Five bandwagon (Widiger et al., 2009; Krueger et al., 2012) identifying dysfunctional dimensions consistent with the Big Five scales.

However, the lexical Big Five personality bastion now seems to have splintered into multiple competing theories proposing six and seven personality dimensions (Saucier, 2009) and even theories with only one, two, and three dimensions (Saucier and Srivastava, 2015). Agreement on parsing the human personality has been very difficult to achieve (Davis and Panksepp, 2018).

Panksepp took a different approach to carving nature at its joints by using electrical stimulation to directly probe the brain for its secrets. He followed in the tradition of Hess (1957) who in the 1930's had evoked a cat rage response using hypothalamic electrical stimulation of the brain (ESB). The basic idea is that if the experimenter introduces a crude unstructured electrical stimulus into a particular brain region and reliably evokes (1) a consistent coherent emotional action pattern and (2) a subjective affect state that can be verified to be pleasant or aversive using self-stimulation or approach-avoidance measures in animals, the ESB has activated an innate, unconditioned, evolutionarily-organized brain circuit linked to the observed emotional behavior (For a summary of humans reporting similar affective shifts following stimulation to homologous brain sites, see Panksepp, 1985). Mainly using ESB—but also pharmacological manipulations and localized brain lesions—Panksepp identified seven emotional brain systems as listed above: SEEKING, RAGE/Anger, FEAR, LUST, CARE, PANIC/Sadness, and PLAY.

These ESB sites are concentrated in subcortical regions of the brain, and correspond with Panksepp's primary-process emotional-action command systems. Cases of such dramatic and unambiguous emotional behaviors or affects have not been elicited from the neocortex. However, more muted displays of laughter have been reported from evolutionarily more ancient cortical areas such as the anterior cingulate cortex (Caruana et al., 2015), which is consistent with the finding that the most striking coherent results with the lowest stimulation required are obtained from evolutionarily ancient subcortical regions such as the periaqueductal gray (PAG).

## Predictions: Primary-Process Emotions and Foundations of Personality and Pathology

One of the ways to verify and extend the validity of a theory is to make predictions and determine whether the evidence confirms the theory. One of Panksepp's predictions was that the primary-process emotions provided the psychobiological foundations of personality. To begin linking the primary emotions to human personality, the Affective Neuroscience Personality Scales (ANPS) were developed (Davis et al., 2003; Davis and Panksepp, 2011). The ANPS was designed to measure self-reported activations of six primary emotions in human lives: SEEKING, RAGE/Anger, FEAR, CARE, PANIC/Sadness, and PLAY (LUST being excluded, as it might limit valid responding and elicit highly socially desirable response patterns). The ANPS has been translated and validated in 10 different languages, and comparisons of the ANPS with Big Five and Five Factor Model personality assessments have uniformly shown close associations of these six emotions to these personality measures (for an overview see Montag and Davis, 2018).

While ANPS PLAY consistently lines up with Big Five Extraversion and SEEKING with Openness to Experience, it is also clear from these studies that some five-factor personality scales are higher-order configurations of the more elemental primary-process emotions. For example, the Big Five/Five Factor Model Agreeableness scale combines the CARE system, which is associated with high levels of Agreeableness, and the RAGE/Anger system, associated with low Agreeableness levels, to conceptually create a “Love-Hate” scale. Further, the Big Five/Five Factor Model Emotional Stability scale places all three of the negatively valenced emotions (RAGE/Anger, FEAR, and PANIC/Sadness) on the low end of Emotional Stability making Emotional Stability a confusing scale to interpret but also conflating the distinctions between these problematic emotions that are so closely linked to the etiology and treatment of psychopathology. These associations are stable across cultures, and the same patterns between individual differences in primary emotional systems as assessed with the ANPS and the Big Five of Personality have been observed in many countries including studies in the USA, Germany and China, potentially hinting at “a global ancestral neuro-biological effect” (Montag and Panksepp, 2017, p.6). For further updates on this issue please see also a recent work by Montag and Panksepp (2018). Also, Montag and Davis (2018) review links between facets of the Big Five of Personality and the ANPS.

Thus, while much work remains to be done linking primary-process emotions to more tertiary language-derived personality models, the initial evidence is that there are close associations between Panksepp's primary-process emotional action systems and the standard Big Five personality assessment with the caveat that the primary emotions offer a more direct biopsychological personality view that more clearly illuminates each of the foundational elements likely constituting our personalities.

An additional note is that no consistent association has been observed in these ANPS studies between the ANPS measured primary emotions and the Big Five Conscientiousness scale. The thought is that Conscientiousness does not measure a primary-process emotion. Rather, it likely measures some aspects of the neocortical inhibition and the resulting cognitive regulation of primary emotions, thus providing tertiary-process top-down control over subcortical emotional reactions (Davis and Panksepp, 2011).

It has been long thought that psychopathology represents extreme expressions of personality characteristics (McDougall, 1908) and that personality disorders and other psychopathologies can be classified on the Big Five/Five Factor Model dimensions (Livesley et al., 1992; Costa and Widiger, 2002). Along these lines, ANPS studies have shown that Panksepp's primal emotions can be used to differentiate Bipolar I and Bipolar II disorders (Savitz et al., 2008a,b) as well as personality disorders (Karterud et al., 2016). See also a newer work investigating the ANPS in multiple sclerosis patients, hence patients with a neurological disorder (Sindermann et al., 2018).

Panksepp has also made several additional predictions about psychiatric treatments that have been tested. Panksepp predicted that autistic children might have dysfunctional brain opioid systems resulting in excess endogenous opioid levels (Panksepp and Sahley, 1987). Experimental research supported his proposal that autism could potentially be treated with low dose naltrexone (an opioid blocker) and suggested that naltrexone benefited a subset of children diagnosed as autistic with as many as 40 percent of autistic children exhibiting enhanced social integration when treated with naltrexone therapy (Bouvard et al., 1995).

Panksepp's work with opioids and their ability to reduce the psychological pain of social separation distress also led him to predict that opioids could reduce suicidal ideation. Research (that was finally able to be conducted in Israel) examined the use of low doses of buprenorphine (a “safe” opioid that has low respiratory depression effects and becomes a mu-opioid blocker at high doses) to counteract suicide in a population of subjects with chronic suicidal thoughts, many of which had already attempted suicide. A small pilot study provided encouraging results (Panksepp and Yovell, 2014). In a larger randomized placebo controlled study using ultra low doses of buprenorphine, there were no instances of suicide. Additionally, there were significantly reduced levels of suicidal thought, and no reports of withdrawal symptoms after the buprenorphine treatment was discontinued at the end of the study (Yovell et al., 2016).

Panksepp has long seen depression as a manifestation of the PANIC/Sadness system, and interestingly, depression was in fact treated with opioids until the 1950s (Bodkin et al., 1995). Panksepp also envisioned that depression could be as much caused by low positive affect as high negative affect (Panksepp et al., 2014). Using the ANPS, Montag et al. (2017) further supported this approach by demonstrating lower SEEKING and higher SADNESS and higher FEAR in depressed patients compared to healthy controls.

A specific treatment prediction was that DBS of the mesolimbic dopamine tract (a main component of the SEEKING system) could alleviate treatment-refractory depression. A German group conducted an early small trial study. The site selected for DBS was the nucleus accumbens, which proved somewhat successful but largely limited to acute effectiveness (Schlaepfer et al., 2008). A larger study involving many of the same investigators showed that five out of ten treatment-resistant depression subjects reached a 50% reduction in their depression ratings (Bewernick et al., 2010). However, in a follow up study electrode placement was moved to the medial forebrain bundle (the most rewarding site in the SEEKING system). In this treatment cohort with treatment-resistant depression, six out of seven patients showed recovery that held through the 33 weeks of the study (Schlaepfer et al., 2013). Although an invasive procedure, this may offer a significant improvement in the quality of life for those with major depression for whom other treatments have failed for many years.

For virtually all drugs used to treat psychopathology, the initial discovery for use in psychiatry had been serendipitous. For example chlorpromazine was first introduced as a presurgical aid for anesthesia before its anti-psychotic benefits were discovered (Ban, 2007). However, a long-developing project based on affective neuroscience research with roots in selecting rats that were more playful than typical (Burgdorf et al., 2005) has yielded a novel drug for treating depression that is proving to be much more effective than anything ever previously used. Identifying juvenile rats that exhibited high levels of frequency-modulated 50 kHz ultrasonic vocalizations during a play experience and subsequently examining their brains for expressed genes, a small group of potential molecules were discovered with the potential for leading to drugs that could successfully treat depression (Burgdorf et al., 2011). Eventually, Glyx13 (this new drug that was later called rapastinel) (Moskal et al., 2017) was given to a group of subjects diagnosed with major depression with remarkable results. Glyx13 elevated moods in 2 h with the positive effects lasting at least seven days with no negative side effects reported (Preskorn et al., 2015). The drug was fast-tracked by the USA FDA, has completed Phase II trials, and is currently in Phase III trials with the FDA. While not yet fully FDA approved, it seems that—as a remarkable validation of cross-species affective neuroscience—we are on the verge for the first time of introducing a psychiatric drug that was developed through an understanding of brain processes. This approach to psychiatry has been a long time coming, but perhaps we should not be surprised by the results.

What predictions are currently being made with a cross-species affective neuroscience perspective? Is it possible that if we just look, multiple fountains of youth for patients with imbalanced emotional brain systems could be discovered in the brain of a playful rat? It might pay to stay tuned to the ongoing work of long time Jaak Panksepp colleagues Joe Moskal and Jeff Burgdorf to see what additional magic they may have up their sleeves (Burgdorf, 2018, personal communication).

## Summary

Jaak Panksepp's career spanned 50 years (Davis and Montag, 2018). Throughout, he worked to provide compelling evidence that animals experienced their emotional arousals: at least all mammals, probably all vertebrates, and with the likelihood that even some invertebrates such as crayfish (separated from humans by perhaps 600 million years) exhibited conditioned preferences to distinct visual environments when associated with injected addictive drugs abused by humans such as amphetamine (Panksepp and Huber, 2004). His was an evolutionary approach that confirmed Darwin's continuity thesis that humans are also animals. He became increasingly committed to the principle that this cross-species affective neuroscience approach would provide the foundational understanding of our subcortical primary-process emotional-affective nature that would be necessary for understanding the neocortical tertiary-level blending of primary and secondary-process values with neocortical analysis and linguistically-based interpretations of experience. Further, animal models taking advantage of the evolutionarily-conserved brain homologies would facilitate the development of novel psychiatric treatments of human emotional imbalances.

Yet, a more cognitive neuroscience-focused climate, convinced that subjective feelings were largely restricted to humans, produced cortical fMRI images of emotional manifestations in humans as their evidence. Panksepp frequently found himself writing that fMRI technology using measures of brain blood flow and oxygenation was better suited to analyzing the larger networks of rapidly firing neocortical cells engaged in cognitive information processing than the physically smaller regions of slower firing subcortical neurons associated with unconditional emotional behaviors and their affects. The more difficult PET imaging seemed a better choice for illuminating subcortical processes (Damasio et al., 2000). However, there have been exceptions with well-designed fMRI studies working with strong emotional arousals highlighting the importance of subcortical structures like the PAG (Mobbs et al., 2007, 2009).

Yet, there remain unresolved debates in the neuroscience community. A major difference reflects Panksepp's position that subcortical activation is sufficient to generate emotions and their affective states that is reviewed above in section “Primary-Process Emotions Survive Decortication”. The cognitive neuroscience counter position is that emotional experience is a “readout” of emotional bodily reactions, which means that cortical perceptions are also necessary. A related dispute emerged from the idea that emotions are learned and “constructed” and elaborated from pleasure and displeasure/arousal dimensions by multiple regions of the brain, whereas Panksepp is clear that primary emotions require no learning and are generated by genetically defined subcortical brain circuits (For a review of additional criticisms see Panksepp et al., 2017).

Yet, Panksepp was eager to reunify emotion theories at the end of his career. Among others, Montag and Panksepp (2016) provided an overview on how Ekman's facial expressions (the view of emotions from the outside) were linked to AN (the view of emotions from the inside). Beyond this—and as a response to constructivism in emotion theories—he wrote: “A primary-process/basic emotion view may prevail in many subcortical regions, and constructivist/dimensional approaches may effectively parse higher emotional concepts as processed by the neocortex. ….In other words, such debates may simply reflect investigators working at different levels of control” (Panksepp, 2010, p. 536).

Whatever the future of affective neuroscience in contributing to our understanding of the human mind from its creative heights to its distressing imbalances, it is heartening that *Frontiers* is publishing a series of articles featuring ongoing affective neuroscience research. Of course many research areas remain to be explored including (1) the interactions of early experience and brain development, (2) gene expression linked to primary-process experience, (3) the relationship between primary emotional experience and memory, (4) how many distinct subcortical emotional circuits exist, (5) whether the neocortex can generate new emotions independent of the subcortical brain, and (6) the capacity of bottom-up subcortical primary-process brain activity to guide cortical development and eventual top-down regulation of primary emotional expression [with additional experimental possibilities discussed in Panksepp et al. (2017)]. It will require many generations of brain scientists to provide satisfactory answers to such questions, which may someday reveal the secrets of the human psyche from personality to psychopathology and perhaps even the origins of consciousness itself.

## Author Contributions

KD wrote the first draft of the manuscript, which was critically revised by CM.

### Conflict of Interest Statement

KD was employed by company Pegasus International. The remaining author declares that the research was conducted in the absence of any commercial or financial relationships that could be construed as a potential conflict of interest.
